# Acceptance and Use of Eight Arsenic-Safe Drinking Water Options in Bangladesh

**DOI:** 10.1371/journal.pone.0053640

**Published:** 2013-01-10

**Authors:** Jennifer Inauen, Mohammad Mojahidul Hossain, Richard B. Johnston, Hans-Joachim Mosler

**Affiliations:** 1 Eawag: Swiss Federal Institute of Aquatic Science & Technology, Department of System Analysis, Integrated Assessment and Modelling, Dübendorf, Switzerland; 2 Eawag: Swiss Federal Institute of Aquatic Science & Technology, Department of Water and Sanitation in Developing Countries (Sandec), Dübendorf, Switzerland; University of Louisville, United States of America

## Abstract

Arsenic contamination of drinking water is a serious public health threat. In Bangladesh, eight major safe water options provide an alternative to contaminated shallow tubewells: piped water supply, deep tubewells, pond sand filters, community arsenic-removal, household arsenic removal, dug wells, well-sharing, and rainwater harvesting. However, it is uncertain how well these options are accepted and used by the at-risk population. Based on the RANAS model (risk, attitudes, norms, ability, and self-regulation) this study aimed to identify the acceptance and use of available safe water options. Cross-sectional face-to-face interviews were used to survey 1,268 households in Bangladesh in November 2009 (n* = *872), and December 2010 (n = 396). The questionnaire assessed water consumption, acceptance factors from the RANAS model, and socioeconomic factors. Although all respondents had access to at least one arsenic-safe drinking water option, only 62.1% of participants were currently using these alternatives. The most regularly used options were household arsenic removal filters (92.9%) and piped water supply (85.6%). However, the former result may be positively biased due to high refusal rates of household filter owners. The least used option was household rainwater harvesting (36.6%). Those who reported not using an arsenic-safe source differed in terms of numerous acceptance factors from those who reported using arsenic-safe sources: non-users were characterized by greater vulnerability; showed less preference for the taste and temperature of alternative sources; found collecting safe water quite time-consuming; had lower levels of social norms, self-efficacy, and coping planning; and demonstrated lower levels of commitment to collecting safe water. Acceptance was particularly high for piped water supplies and deep tubewells, whereas dug wells and well-sharing were the least accepted sources. Intervention strategies were derived from the results in order to increase the acceptance and use of each arsenic-safe water option.

## Introduction

Arsenic contamination of drinking water resources is increasingly recognized as a global health problem. The chronic ingestion of arsenic has been linked to internal cancers [1] and elevated mortality rates from myocardial infarction [2], along with numerous other health problems. Nowhere is the problem more serious than in Bangladesh. Arsenic exposure has been estimated to account for 21% of all mortality in one moderately contaminated sub-district of Bangladesh [3] and for at least 24,000 deaths per year nation-wide [4].

The arsenic problem was first recognized in the 1990s, when a national survey showed that approximately 27% of shallow tubewells exceeded Bangladesh’s permissible arsenic limit of 50 µg/L, while 46% exceeded the WHO’s provisional guideline value of 10 µg/L [5]. Cities and municipalities, for the most part, supply water from deep, arsenic-free aquifers; however, in rural and peri-urban areas, shallow, privately-owned tubewells are the principal sources of drinking water. Early mitigation efforts focused on raising awareness of the risks posed by arsenic, which was a daunting challenge because arsenic has no taste or odor and symptoms take years to develop. Basic information was imparted to villagers during a massive tubewell screening campaign from 2000 to 2006 in which nearly 5 million wells in arsenic-prone areas were tested and painted red or green, depending on whether they were over or within the national standards [6].

Subsequent efforts focused on the promotion and installation of alternative arsenic-free water sources. The National Arsenic Mitigation Policy recommends that wherever feasible, piped water systems should be promoted and that preference be given to surface water over groundwater sources [7]. The implementation plan accompanying the policy endorsed the promotion of various alternative water sources. Improved dug wells (hand dug shallow wells, generally around 1 m in diameter and 10–20 m in depth, with sanitary protection at the surface) and pond sand filters (small community slow sand filters) were to be given priority, while deep tubewells (drilled wells, generally 1.5 inches in diameter and >150 m in depth, with sanitary protection and a handpump at the surface) were to be installed only as a last resort. Piped water systems were identified as the long-term goal. Other endorsed alternatives included large-scale surface water treatment plants, rainwater harvesting systems, and household or community arsenic removal technologies. These are described in more detail in the implementation plan [8]. The technical suitability of these alternatives depends on local hydrogeologic and geographic conditions, so different options are promoted in different zones of the country.

The local-scale spatial distribution of arsenic is highly variable [9], and in many affected villages, there are enough safe shallow tubewells to supply the entire population [10]. Although the policy and implementation plan do not explicitly refer to the sharing of safe, shallow tubewells, well-sharing (sometimes referred to as well-switching) was a key message in the tubewell screening program.

By 2006, it was estimated that more than 100,000 alternative sources had been installed in arsenic-affected areas. In spite of the stated policy preference for surface water, 70% of new installations were deep tubewells [11]. Localized studies showed that well-sharing was also common in some areas [6], [12]. By 2006, an expert review estimated that 29% of the population initially exposed to arsenic had switched to arsenic-safe shallow tubewells and that another 12% had switched to deep tubewells [13]. Use of the other alternatives was considered negligible.

However, the allure of the arsenic-contaminated shallow tubewells is strong, especially as memories of the well-screening survey fade, and there is a lack of data about long-term water use practices. All safe water options involve more time or effort, and collecting water from a community source is a very different behavior than using one’s own private tubewell. Operation and maintenance may be more complicated, and community-level management can be erratic. In a survey of 1,000 arsenic-safe water sources, Kabir found that 10% of deep tubewells, nearly a quarter of dug wells and pond sand filters, one-third of rainwater harvesting systems, and 83% of arsenic removal technologies were non-functional [11]. However, this review focused on technical performance, and little information is available regarding end-user acceptance or factors influencing families to use or refrain from using arsenic-safe water sources. Of the few studies in this domain, most have been concerned with people’s knowledge and risk awareness (e.g. [14]), even though increased knowledge often does not translate into the use of arsenic-safe water options [13]. Research that considered further acceptance factors has shown that the use of available arsenic-safe water options is indeed related to distance [12], [15]. Other identified factors are perceived taste [16] and social barriers for women [15]. However, due to the lack of theoretical background, these studies investigated only a few possibly influential acceptance factors. In an attempt to overcome this, a study of deep tubewell use in Sreenagar, Bangladesh used psychological analysis derived from the Protection Motivation Theory to show that social factors were much more important determinants of water source usage than awareness of arsenic or perceived vulnerability and severity [17]. While this study provided important first insights into the acceptance and use of deep tubewells, clearly more knowledge is needed about the other arsenic-safe water options, and their comparison.

The aim of the present study is to provide an update on the use of available arsenic-safe water options and to investigate a comprehensive selection of social and psychological acceptance factors of water options. We address two main questions:

1. To what extent are available safe water options actually used by people in contaminated areas?
2. Which safe water options are more accepted than others regarding psychological factors, both for users and non-users?

To determine the acceptance of an option, we drew on the RANAS model [18]. In this model, psychological factors are ordered in five different blocks: Risk, Attitudinal, Normative, Ability, and Self-regulation factors. These blocks are comprised of several psychological factors, which represent a compilation of the possible drivers of health behavior change [19]. We use the term ‘acceptance’ as a comprehensive construct to describe positive values in psychological factors that are influencing the use of a certain option. High acceptance means that this option has high values in several psychological factors of the RANAS model.

In the present study, we focus on the differences between the options regarding these acceptance factors. Thereby, insight will be gained on which options are more accepted than others and which acceptance factors have to be taken into account when introducing a certain option, and to ensure its sustained use.

## Methods

### Participants and Procedures

Data were collected during two cross-sectional surveys in rural Bangladesh, with a total sample size of 1,268 households. Participants were at risk of drinking arsenic-contaminated water (i.e., they either owned a contaminated tubewell or collected water from one) and had access to one of the following arsenic-safe water options: dug wells, pond sand filters, deep tubewells, piped water supply, household arsenic removal, community arsenic removal, household rainwater harvesting systems, or well-sharing.

In November 2009, a survey (N = 872) of 30 days duration was conducted in six districts of Bangladesh: Satkhira, Khulna, Bagerhat, Comilla, Munshiganj, and Brahmanbaria. These districts were selected due to their high levels of average arsenic contamination. In all of our study locations, people had access to one of seven arsenic-safe water options: dug wells, pond sand filters, piped water supply, household arsenic removal filters, community arsenic removal filters, household rainwater harvesting, and well-sharing. Due to hydrogeological or geographic conditions, in most of these areas only one or two safe water options were actively promoted. Mitigation options, with the exception of well-sharing, were identified through discussions with the Department of Public Health Engineering (DPHE), UNICEF, and local governments. These options were installed through either a DPHE/UNICEF or NGO arsenic mitigation project, between 1 and 5 years prior to the survey. In these mitigation projects, beneficiaries were educated about arsenic contamination, involved in site selection for alternative sources and normally paid from 10–20% of the capital costs. Well-sharing was not formally promoted, so a different selection process was followed. Within the same regions as the seven mitigation options, areas with moderate contamination density but without known mitigation options were identified with the help of DPHE and local authorities, on the basis of prior screening campaigns. Households who lived within walking distance of an arsenic-safe, green-marked shallow tubewell were considered potential well-sharers. If they owned or had previously collected drinking water from an arsenic-contaminated well but now collected water from arsenic-safe wells, they were considered well-sharers. In turn, households who did not make use of this alternative were considered non-users of well-sharing.

The second study was conducted in the Comilla district during two weeks in December 2010, where interviewees had access to an eighth option: arsenic-safe deep tubewells (N = 396), installed through a DPHE/UNICEF project.

The survey was carried out by professional Bangladeshi interviewers. Conducting structured psychological surveys in rural areas of developing countries is always a challenge. We therefore devoted much time and effort to interviewer training, including extensive rehearsals of interviewing techniques and how to convey knowledge regarding arsenic contamination, arsenic-safe water options, and basic health-behavior theory. Quality control was ensured by the first and second author, a master’s student, and two local supervisors.

In the villages, interviewers selected households by random-route sampling [20]. Interviewers first screened whether the household met inclusion criteria. The participants were the persons responsible for water collection for the selected households. After receiving informed consent, interviewers then conducted structured interviews regarding the arsenic-safe water options that the participants had access to. The duration of the interviews ranged from 1 to 1.5 hours. The rate of refusal was low, which is quite common for research in developing countries (e.g. [17]). The only exception was owners of household arsenic removal filters: 30% declined to participate.

### Ethics Statement

This study was conducted in strict compliance with the ethical principles of the American Psychological Association (APA) and the Declaration of Helsinki. It underlies the ethics review board of the ETH, Swiss Federal Institute of Technology Zurich. This review board exempts survey studies that do not comprise an intervention from obtaining ethical approval: “Alle Forschungsuntersuchungen am Menschen … müssen vor Versuchsbeginn durch die Ethikkommission der ETH Zürich beurteilt werden … Reine Befragungen im Sinne von Meinungsumfragen sind keine Forschungsuntersuchungen am Menschen [All research projects involving human participants … must be reviewed by the ethics review board of the ETH Zürich prior to commencement … Pure survey research, i.e. opinion surveys are not considered as research involving human participants]” (http://www.vpf.ethz.ch/about/commissions/EK).

Oral informed consent was obtained from all study participants. (Written consent was not obtained due to the high rate of illiteracy.) Whenever a selected household refused to participate in the study, the interview was ended immediately. The number of refusing households was marked in a dedicated space in the questionnaire of the next consenting household. The regulations of the ethics review board of the ETH allow for either oral or written consent without preference for either form.

### Measures

A structured questionnaire was specifically developed for this study to assess water consumption and the acceptance factors from the RANAS model (see Table 1 for definitions of all constructs and their operationalizations in the present study). Furthermore, sociodemographic characteristics were assessed. The questionnaire was translated into Bengali and then back-translated into English to verify the quality of the translation. During the questionnaire preparation and pretesting, we worked closely with local collaborators who advised us on how to formulate the questions and answers in a way that participants could best understand.

**Table 1:** Psychological factors and their assessment.

Psychological factors	Definition	Assessment question
*Risk factors*		
Perceived vulnerability [21]	A person’s subjective perception ofhis/her risk of contractingarsenicosis	“How high or low do you feel are the chances that you get arsenicosis?” (−4 = very low to 4 = very high)
Perceivedseverity [21]	A person’s perception of the seriousness of the consequences of contracting arsenicosis	“Imagine that you contracted arsenicosis, how severe would be the impact on your life in general?” (0 = not at all severe to 4 = very severe)
Factualknowledge [22]	An understanding of how a person could become affected by arsenic	2009: Seven items assessed factual knowledge. Respondents were asked to describe what arsenic is, to name the effects that arsenic can have on the body, to name causes of the effects of arsenic on the body, and to give an estimate how long it takes for arsenic to take effect on the body. Three further questions asked whether arsenic was contained in water from red (i.e., arsenic-contaminated) tubewells or in food cooked with that water and if water from the arsenic-safe option the respondents used was free of arsenic. 2010∶14 questions asked about which water sources contained arsenic, whether contaminated water was safe to drink, which medical conditions could be caused by arsenic, and for which tasks it was okay to use arsenic-contaminated water. (0 = no knowledge to 4 = maximum knowledge)
*Attitude factors*		
Instrumentalbeliefs [23]	How time-consuming is collection	“Do you think that collecting water from arsenic-safe option is time consuming?” (0 = not at all time consuming to 4 = very time consuming).
Affective beliefs [24]	Taste and temperature	“How much do you like or dislike the taste (temperature) of the water from the arsenic-safe water option?” (- 4 = dislike it very much to 4 = like it very much).
*Normative factors*		
Descriptivenorm [25]	Perceptions of which behaviors are typically performed	“How many people outside your family collect water from arsenic-safe option?” (0 = almost nobody to 4 = almost everybody)
Injunctivenorm [26]	Perceptions of which behaviors are typically approved or disapprovedof by important others	“You drink water from the arsenic-safe option. Do people who are important to you rather approve or disapprove of this?” (−4 = they disapprove very much to 4 = they approve very much)
*Ability factors*		
Self-efficacy[27]	The belief in one’s capabilities to organize and execute the course of actions required to manageprospective situations	“How sure are you that you can collect as much water from the arsenic-safe option as you need?” (0 = not at all sure to 4 = very sure)
Actionknowledge [22]	Knowing how to perform thebehavior	2009: Participants were asked to describe how arsenic and its harmful effects can be avoided, and to name as many arsenic-safe water options as they knew. 2010: Respondents were asked whether they knew the location of a safe water option in their village, whether it was safe to drink from a green-colored tubewell, whether arsenic can be removed by boiling, and to name water sources that are free from arsenic.
*Self-regulation factors*		
Copingplanning [28]	How the person plans to cope with distractions and barriers	“Have you made a detailed plan regarding what to do when the arsenic-safe water option gets broken?” (0 = no detailed plan at all to 4 = very detailed plan)
Remembering[29]	The behavior needs to beremembered at critical moments	“How often does it happen that you forget to go to collect water from the arsenic-safe option?” (0 = almost never to 4 = almost always)
Commitment[30]	How committed the person is tothe new behavior	“Do you feel committed to collect water from the arsenic-safe option?” (0 = not at all to 4 = very much)

Water consumption was assessed by asking people how many vessels of which water option and in total they collected for drinking on a typical day.

Questions used to assess the psychological factors are described in Table 1. Most factors were scored on a 0 to 4 scale, representing the minimum and maximum possible values. Factors that could have negative as well as positive impacts (e.g., “How much do you dislike or like the taste of water?”) were scored on a scale of −4 to 4.

In most cases, a single question was used to quantify each factor, but ‘factual knowledge’ and ‘action knowledge’ were both determined through a series of questions. In the 2009 survey, knowledge was assessed through open-ended questions; for the 2010 survey, closed-ended questions were used. Each correct answer was assigned one point. This was transformed into the value range of the other variables to standardize the ranges (0 = no knowledge to 4 = maximum knowledge).

In addition, open questions were asked in order to provide a more detailed insight into people’s beliefs. Regarding vulnerability, participants were asked why they felt vulnerable to developing arsenicosis or not. Furthermore, answers to open questions about the advantages and disadvantages of collecting water from the arsenic-safe water options, and whether there were any seasonal peculiarities in collecting water from the safe option, provided a deeper understanding of what people liked or disliked about water taste and temperature.

We also assessed socio-demographic parameters: gender, literacy, religion, age, number of people living in the household, household income, and years of formal education. Finally, respondents were asked whether and how much money their household had contributed to installing the arsenic-safe water option and how much they paid every month to use it.

### Data Analysis

Data were analyzed using SPSS 18.0. All analyses were conducted for users and non-users of arsenic-safe water options separately. For scaled items, means and standard deviations were computed separately for non-users and users for each of the arsenic-safe water options. For dichotomous items, percentages were calculated. First, non-users and users for the entire sample were compared regarding all acceptance factors with independent-sample t-tests. Then, to investigate which options were rated high or low regarding each acceptance factor, the frequencies of each option were compared to the overall frequencies (means or percentages) of the entire sample by one-sample t-tests and Pearson χ^2^ tests, respectively. In accordance with the RANAS model, acceptance of a particular option will be higher the more of its acceptance factors exceed the overall sample average.

## Results

The characteristics of the study participants can be found in Tables 2 and 3. Note that bolded values are significantly higher, and italicized values are significantly lower than the values of the overall sample (*p*<.05).

**Table 2:** Demographic characteristics of participants by users and non-users of the available arsenic-safe water option. (*M* = mean, *SD* = standard deviation).

		Overall^1^		Piped water supply		Deep tubewells		Pond sand filters		Community arsenic-removal		Dug wells		Well-sharing		Rainwater harvesting		Household arsenic-removal	
		*M*	*SD*	*M*	*SD*	*M*	*SD*	*M*	*SD*	*M*	*SD*	*M*	*SD*	*M*	*SD*	*M*	*SD*	*M*	*SD*
Age	Non-users	37.5	12.8	39.9	10.5	36.3	13.0	40.7	14.4	38.3	12.7	36.0	11.9	*32.9* *	11.2	**40.3** *	12.4	35.3	12.4
	Users	36.8	12.3	38.9	14.0	35.6	11.7	38.3	12.2	36.3	11.9	36.4	11.7	36.1	12.0	**40.2** *	10.6	36.3	12.9
No. of people living in household	Non-users	5.4	2.3	5.1	1.9	5.6	2.5	5.1	2.1	5.8	2.8	5.1	1.9	5.6	2.5	5.3	1.9	6.1	2.8
	Users	5.5	2.3	5.6	2.1	5.7	2.3	5.6	2.5	5.2	2.4	5.5	2.3	5.3	2.4	*4.9* *	1.6	5.8	2.2
Monthly income (BDT)	Non-users	8935	8352	12647	13214	**10939** *	8986	*5360* *	2841	*6317* *	4782	*4961* *	2985	*5450* *	3291	**12355** *	10969	8833	5303
	Users	9648	7840	**11931** *	9007	**11480** *	8879	*7373* *	5435	*7439* *	5959	*6837* *	4494	*7439* *	4542	**12237** *	6403	9207	9019
Education (years)	Non-users	5.0	4.1	*2.1* *	3.3	**5.7** *	3.7	4.6	4.6	*2.6* *	3.2	5.2	4.0	5.6	4.8	4.9	4.2	4.3	3.7
	Users	5.0	4.1	*4.1* *	3.4	5.2	3.7	5.8	4.3	4.4	3.8	4.4	4.1	5.6	4.2	5.9	4.5	4.7	4.6

*Note.* For each row, comparisons between each option and the overall sample were computed: **Bolded values** are significantly greater than the overall means. *Italicized values* are significantly lower than the overall means.

1 In this column, T-Tests were computed to compare users and non-users in the overall sample regarding their age, number of people living in household, monthly income, and education. None of the comparisons achieved significance (*p*>.05).

* *p*<.05.

**Table 3:** Numbers and proportions of users and non-users of the available arsenic-safe water option.

		Overall^1^		Piped water supply		Deep tubewells		Pond sand filters		Community arsenic-removal		Dug wells		Well-sharing		Rainwater harvesting		Household arsenic-removal	
		*N*	*%*	*n*	*%*	*n*	*%*	*n*	*%*	*n*	*%*	*n*	*%*	*n*	*%*	*n*	*%*	*n*	*%*
Number of users and non-users of the arsenic-safe water option	Non-users	480	37.9^2^	18	*14.4* *	182	**46.0** *	60	**48.4** *	33	*26.4* *	64	**51.6** *	36	*28.8* *	78	**63.4** *	9	*7.1* *
	Users	788	62.1^2^	107	**85.6** *	214	*54.0* *	64	*51.6* *	92	**73.6** *	60	*48.4* *	89	**71.2** *	45	*36.6* *	117	**92.9** *
Households paid for installing the arsenic-safe option (% yes)^3^	Non-users	75	15.6	4	22.2	5	*2.8* *	0	*0.0* *	1	*3.0* *	4	*6.3* *	0	*0.0* *	55	**73.3** *	6	**100** *
	Users	346	43.9	70	**65.4** *	73	*36.5* *	11	*17.2* *	14	*15.2* *	25	41.7	0	*0.0* *	36	**81.8** *	117	**100** *
Households that pay to use thearsenic-safe option (% yes)	Non-users	–	–	–	–	–	–	–	–	–	–	–	–	–	–	–	–	–	–
	Users	197	25.0	104	**97.2** *	2	*0.9* *	5	*7.8* *	83	**90.2** *	3	*5.0* *	0	*0.0* *	0	*0.0* *	0	*0.0* *
Gender (% female)	Non-users	382	79.6	15	**83.3** *	180	**98.9** *	40	*66.7* *	24	72.7	39	*60.9* *	21	*58.3* *	54	*69.2* *	9	**100** *
	Users	626	79.4	79	73.8	210	**98.1** *	43	*67.2* *	63	*68.5* *	43	71.7	59	*66.3* *	35	77.8	94	80.3
Literacy rate	Non-users	317	66.0	6	*33.3* *	140	**76.9** *	36	60.0	13	*39.4* *	41	64.1	22	62.9	53	67.9	6	66.7
	Users	526	66.8	68	65.4	151	70.6	45	73.8	56	63.6	36	61.0	64	73.6	36	81.8	70	61.4
Religion (% muslim^4^)	Non-users	457	95.2	18	100.0	180	**98.9** *	47	*78.3* *	33	100.0	64	100.0	36	100.0	74	94.9	5	*55.6* *
	Users	705	89.5	107	**100.0** *	200	93.5	39	*60.9* *	79	85.9	56	93.3	83	93.3	38	84.4	103	88.0

*Note.* For each row, comparisons between each option and the overall sample were computed: **Bolded values** are significantly greater than the overall frequencies. *Italicized values* are significantly lower than the overall frequencies.

1 In this column, Chi-Square tests were computed to compare users and non-users in the overall sample regarding proportion of households who paid for installing the safe option (*p*<.05), gender (not significant [n.s.]), literacy (n.s.), and religion (*p*<.05).

2 One-dimensional Chi-Square test (Null hypothesis: Equal count of users and non-users): *p*<.001.

3 Due to missing responses, valid percents are reported. Some participants did not know whether their household had paid to install the safe option.

4 All other participants reported Hinduism as their religion.

* *p*<.05.

Table 2 shows that the demographic characteristics of the different groups interviewed were broadly similar, although income was regionally variable: It was higher in areas surveyed for rainwater harvesting and deep tubewell use and lower in areas surveyed for pond sand filters, dug wells, and well-sharing. Comparisons of users and non-users in the overall sample yielded no significant differences in income or any of the other demographic characteristics. A multivariate analysis of variance of income, confirming the univariate analyses, yielded a significant main effect for arsenic-safe water option (*F* [7, 1080] = 14.16, *p*<.001), but no significant effects for user/non-user of safe water nor for the water option X user/non-user interaction.

### Use of Arsenic-safe Water Options

Overall, nearly two-thirds of households (62.1%) were using the available arsenic-safe water options for drinking at the time of the survey (Table 3). The most used options were household arsenic removal filters, piped water supply, community arsenic removal, and well-sharing. In contrast, deep tubewells, pond sand filters, and dug wells were used by approximately half the people who had access to these options. Finally, only one third of households used available rainwater harvesters.

Significantly more users made a financial contribution to installing an available safe water option in comparison to non-users (*χ^2^* = 108.70; *p*<.001). Contributions ranged from 10 Bangladeshi Taka (BDT; exchange rate was approximately 77 BDT per US dollar) to 35,000 BDT, with a median of 700 BDT. All users and non-users had contributed to paying for their household arsenic filters. Also, most people had contributed to installing their rainwater harvesters or piped water systems. Regarding monthly payments, with few exceptions, the only options that people paid for using were community arsenic removal (*M = *16.0 BDT, *SD* = 12.4 BDT) and piped water supply (*M = *62.0 BDT, *SD* = 24.5 BDT).

### Factors of Acceptance for Eight Arsenic-safe Water Options

All psychological factors presented in Table 4 were significantly differentiated between non-users and users of arsenic-safe water options (*p*<.05). However, some differences were very small, i.e., regarding severity, factual, and action knowledge. Users reported high severity. However, in contradiction with the theoretical assumptions, their vulnerability to developing arsenicosis was low. Water taste and temperature were rated particularly high, but they reported that collecting water was somewhat time-consuming. Users also reported high injunctive norms, medium descriptive norms, high self-efficacy, quite detailed coping planning, and a strong commitment to using safe options.

**Table 4:** Perceived risk, attitudes, norms, abilities, and self-regulation (*M* = mean, *SD* = standard deviation) by users and non-users of the available arsenic-safe water option.

		Overall^1^		Piped water supply		Deep tubewells		Pond sand filters		Community arsenic-removal		Dug wells		Well-sharing		Rainwater harvesting		Household arsenic-removal	
Psychological factors		*M*	*SD*	*M*	*SD*	*M*	*SD*	*M*	*SD*	*M*	*SD*	*M*	*SD*	*M*	*SD*	*M*	*SD*	*M*	*SD*
***Risk factors***																			
Severity	Non-users	3.20	0.74	*2.72* *	0.75	3.24	0.58	**3.48** *	0.70	3.39	0.70	3.09	1.00	3.25	0.87	*2.97* *	0.70	3.11	0.78
	Users	3.40	0.67	3.45	0.66	3.44	0.57	3.48	0.53	3.45	0.58	**3.60** *	0.64	*3.13* *	0.77	3.27	0.81	3.34	0.77
Vulnerability	Non-users	0.78	2.04	0.11	1.97	**1.15** *	2.18	0.22	2.20	0.73	1.79	1.16	1.88	0.47	2.41	0.46	1.45	0.00	1.66
	Users	−2.28	1.85	−*2.75* *	1.64	−2.18	2.07	−2.45	1.72	−2.34	1.59	−*1.50* *	2.27	−2.01	1.77	−2.33	1.76	−2.49	1.54
Factual knowledge	Non-users	1.73	0.61	1.71	0.59	**1.85** *	0.54	1.57	0.66	1.68	0.57	*1.25* *	0.64	1.69	0.61	**2.02** *	0.45	1.91	0.27
	Users	1.93	0.47	1.90	0.44	1.95	0.48	1.91	0.44	1.92	0.44	*1.73* *	0.52	1.90	0.47	1.95	0.52	**2.03** *	0.44
***Attitude factors***																			
Taste	Non-users	1.96	1.81	2.50	1.10	**2.48** *	1.46	1.43	2.18	1.33	2.12	*1.05* *	2.24	1.42	1.81	2.22	1.29	2.33	1.66
	Users	3.11	1.16	**3.55** *	0.60	3.21	0.90	2.95	1.23	2.78	1.65	*2.40* *	2.10	*2.85* *	1.02	3.17	0.74	**3.40** *	0.63
Temperature	Non-users	1.96	1.67	**2.83** *	0.79	**2.66** *	1.15	*1.07* *	1.86	*1.06* *	2.15	*1.20* *	1.87	1.47	1.72	2.16	1.27	0.67	2.29
	Users	2.77	1.41	**3.20** *	1.25	**3.15** *	0.86	2.77	1.03	*2.04* *	1.92	*2.22* *	2.09	*2.48* *	1.32	2.82	1.30	2.72	1.44
Time consuming	Non-users	2.38	1.20	2.06	0.94	**2.83** *	1.03	**2.77** *	1.17	2.61	1.03	2.16	1.13	2.44	1.00	*1.13* *	0.94	2.56	0.73
	Users	1.52	1.04	*1.14* *	1.00	1.47	1.10	1.56	1.05	1.48	1.02	**1.88** *	1.03	**1.96** *	0.85	*1.09* *	0.87	1.59	0.98
***Norm factors***																			
Descriptive norm	Non-users	1.11	0.89	1.39	0.70	1.07	0.89	1.23	0.74	1.45	0.97	1.27	1.04	**1.42** *	0.77	*0.64* *	0.72	0.89	0.78
	Users	2.28	1.06	**2.60** *	0.86	**2.72** *	1.18	2.42	0.91	**2.59** *	0.84	*1.97* *	0.78	*1.78* *	0.81	*1.22* *	0.67	*1.82* *	0.98
Injunctive norm	Non-users	2.40	1.80	2.61	1.09	2.66	1.83	2.60	1.73	**3.00** *	1.52	2.30	1.84	*1.56* *	2.03	*1.79* *	1.69	2.67	1.41
	Users	3.13	1.36	**3.54** *	0.70	3.02	1.54	3.20	1.12	3.27	1.12	3.17	1.61	*2.31* *	1.72	2.82	1.71	**3.54** *	0.71
***Ability factors***																			
Action knowledge	Non-users	1.99	0.99	*1.50* *	0.34	**2.94** *	0.78	*1.21* *	0.60	*1.29* *	0.57	*1.46* *	0.53	*1.39* *	0.63	*1.51* *	0.52	1.78	0.67
	Users	1.87	0.87	*1.50* *	0.64	**2.83** *	0.75	*1.15* *	0.46	*1.41* *	0.47	*1.45* *	0.48	*1.44* *	0.00	1.74	0.53	1.77	0.65
Self-efficacy	Non-users	1.37	1.15	**2.50** *	1.04	*1.18* *	1.09	*0.93* *	0.99	1.45	1.03	1.52	1.15	1.33	1.10	1.56	1.24	**2.78** *	0.44
	Users	3.27	0.86	**3.68** *	0.65	*3.10* *	0.88	3.42	0.73	3.27	0.65	*2.83* *	1.17	*2.94* *	1.02	3.27	0.89	**3.62** *	0.55
***Self-regulation factors***																			
Coping planning	Non-users	0.97	0.96	**2.39** *	0.78	*0.63* *	0.75	0.95	1.10	1.15	0.91	1.11	0.91	1.11	0.95	1.06	0.93	**2.11** *	0.78
	Users	2.06	1.06	**2.44** *	0.90	*1.89* *	1.05	2.08	1.06	1.89	0.98	*1.76* *	1.01	*1.69* *	1.06	2.04	1.04	**2.60** *	1.03
Remembering	Non-users	−	−	−	−	−	−	−	−	−	−	−	−	−	−	−	−	−	−
	Users	0.26	0.56	0.21	0.53	**0.40** *	0.70	0.20	0.54	0.20	0.43	0.43	0.77	0.19	0.40	0.00	0.00	*0.18* *	0.43
Commitment	Non-users	1.42	1.18	**2.06** *	0.94	*1.06* *	1.05	1.62	1.12	1.24	1.20	1.63	1.34	*0.97* *	1.11	**2.06** *	1.11	1.44	0.88
	Users	3.13	0.89	**3.60** *	0.53	3.02	0.90	3.28	0.74	3.17	0.81	*2.68* *	1.32	*2.79* *	0.95	3.00	0.77	**3.30** *	0.72

*Note:* For each row, comparisons between each option and the overall sample were computed: **Bolded values** are significantly greater than the overall means. *Italicized values* are significantly lower than the overall means.

1 In this column, T-Tests were computed to compare users and non-users in the overall sample regarding each acceptance factor. All comparisons achieved significance (*p*<.001, except for action knowledge: *p*<.05).

* *p*<.05;

*** *p*<.001.

Non-users showed higher vulnerability than users, with high standard deviations, which indicated different types of non-users: some felt quite vulnerable to developing arsenicosis, while others did not. In comparison to users, non-users also showed lower taste and temperature ratings and reported that collecting water from the safe option was quite time-consuming. Furthermore, non-users displayed lower norms, much lower self-efficacy and coping planning, and much lower commitment to using safe water options.

Answers to an open question yielded deeper insights into the counterintuitive result that users of safe water options felt less vulnerable to developing arsenicosis than non-users. Of all the users of safe options, 639 (81.1%) did not feel vulnerable. The vast majority of them reported that this was due to the fact that they were drinking arsenic-safe water (633; 99.1%). In turn, 274 (57.1%) of all non-users felt vulnerable. Most of them reported that the reason for this was that they drank from arsenic-contaminated (218; 79.6%) or untested tubewells (46; 17%). Finally, 84 (18%) non-users did not feel vulnerable. Of these participants, 30 (36%) reported that this was due to their occasionally drinking arsenic-safe water or that they had not encountered any problems with the water, even though they had been drinking it for a long time.

The answers to open questions about water taste and temperature were insightful. First of all, the vast majority of participants liked the taste of their drinking water to some extent (1,144 with taste ratings >0). Only 59 respondents disliked the water from the safe option, and most of them were people with access to dug wells (22; 37%). Of the people who disliked the water’s taste, many reported sandy (25%) and iron tastes (25%), as well as bad smells (33%). Participants who liked the taste, however, were not able to clearly describe why. The most frequent answers were that the water had no bad smell (67; 9%) and contained less iron (48; 6%).

Regarding temperature, as with the taste ratings, only a few respondents disliked this (55; 4%). Many of them were households with access to community arsenic removal filters (29%) or dug wells (26%), as well as household filter owners (13%) and people with access to pond sand filters (13%). Some of the respondents who disliked the water temperature reported that the water was too cold in the winter (26%) or too hot in the summer (26%). Again, as with taste, people who liked the water temperature did not have a clear reason as to why they did.

The following section presents the results on the acceptance of each arsenic-safe water option. For this purpose, the ratings of each acceptance factor for a particular option was compared with the overall sample mean.

### Most Accepted Water Options: Piped Water Supply and Deep Tubewells

Piped water supply and deep tubewells were exceptionally well-supported by the psychological factors (Table 4). Users of piped water reported significantly above-average ratings for taste, and rated collecting safe water as below-average in terms of time-consumption than the average study participant. Both users and non-users had significantly higher water temperature ratings. Users also displayed above-average social norms. Furthermore, people with access to piped water also displayed above-average self-efficacy, more detailed coping plans, and higher levels of commitment.

Similarly, participants with access to deep tubewells gave higher temperature ratings. Non-users, however, rated collecting water from deep tubewells as above-average in terms of being time-consuming. Generally, households with access to deep tubewells displayed higher levels of descriptive norms and above-average action knowledge. In contrast to households with access to a piped water supply, however, they had below-average self-efficacy and less detailed coping plans. Finally, non-users displayed below-average levels of commitment.

### Least Accepted Water Options: Dug Wells and Well-sharing

Dug wells and, to a lesser degree, well-sharing were poorly supported by psychological factors. Households with access to dug wells showed significantly lower levels of knowledge compared to other households, as well as the lowest ratings for water taste and temperature. Furthermore, compared to the average safe water user, users of dug wells perceived collecting water as more time-consuming and displayed lower levels of descriptive norms, self-efficacy, coping planning, and commitment.

Households who used neighboring safe tubewells (well-sharers) reported below-average taste and temperature ratings, and found collecting water more time-consuming. Well-sharers also reported lower descriptive norms than the average study participant, whereas for potential well-sharers (households who do not use available neighboring safe wells), the opposite was true. Both current and potential well-sharers rated significantly lower in terms of injunctive norms. Furthermore, self-efficacy and coping planning were below average for well-sharers. Finally, commitment was low for both actual and potential well-sharers.

### Moderately Accepted Options: Community and Household Arsenic Removal, Rainwater Harvesting, and Pond Sand Filters

Community arsenic removal and pond sand filters were both rated average on most factors. The exception for community arsenic removal was water temperature: users and non-users were significantly less satisfied than the average study participant. Similarly, non-users of available pond sand filters were significantly less satisfied with water temperature. Furthermore, they found collecting water significantly more time-consuming and were rated significantly lower in self-efficacy than the average non-user in the study.

Users of household filters displayed the highest levels of factual knowledge. Also, water from household filters received the highest taste ratings from users. However, descriptive norms for this option were low, which was also found to be the case for the other household options (i.e., rainwater harvesting and well-sharing). Users and non-users of household arsenic removal filters displayed above-average levels of self-efficacy and coping planning. Finally, users displayed low levels of remembering, but the highest levels of commitment.

Rainwater harvesting was rated as the least time-consuming of all safe options. However, rainwater harvesting was rated as below-average in terms of the descriptive norms. Non-users also reported lower injunctive norms.

## Discussion

The aim of the present study was to determine the acceptance and use levels of eight available arsenic-safe water options in Bangladesh. Knowledge of the status quo of people’s acceptance and use of these options will provide a starting point for developing interventions to enhance their sustained use and can also guide experts in making choices regarding which options to implement.

A major finding of this study was that one third of households in Bangladesh who are at risk of drinking arsenic-contaminated water, and who have access to one of the eight arsenic-safe water options, do not use these options. Some options are used by as little as one-third of those who could make use of them. This implies that the number of people at risk of developing arsenicosis in Bangladesh may be underestimated [6]. Refining behavior-change campaigns is an essential step in improving the acceptance and use of the available safe water options. However, technical improvements to safe water technologies may also be in order. The present study provides insights into people’s acceptance. Importantly, in line with previous research on safe water consumption [31], [32], it was shown that the RANAS factors reliably differentiate between users and non-users of arsenic-safe water options. In the following, we will discuss the psychological factors that were found favorable or unfavorable for the acceptance of each safe water option (Table 4), as well as possible interventions to increase acceptance.

Users of **piped water supply** showed a high degree of acceptance; the vast majority of the related behavioral factors were well above the average of all options. This result is in line with Hoque et al., who also found high acceptance of piped water [15]. Additionally, users of piped water rate this option as being not very time-consuming and perceive low levels of vulnerability when they drink this water. This finding supports the increased installation of piped water supplies in arsenic-affected areas. However, piped water systems often fail because of technical, institutional, or financial difficulties. Tariff collection, for example, often presents a problem, especially where local administrations have limited capacity. Still, piped water is recognized as a long-term strategic goal by the government. It can be expected that as more experience is gained with this technology in Bangladesh, these challenges will be overcome.

**Deep tubewells** also displayed high acceptance scores, but are rated as being time-consuming, which, in line with the study by Mosler et al. [17], seems to be an issue that people find difficult to cope with. Non-users find this particularly hindering, which is perhaps why their commitment to collecting water from deep tubewells is below average. This might be a reason why only slightly more than half of respondents with access to deep tubewells actually use them. This perception may be changed by adding positive feelings to collecting deep tubewell water, e.g., by promoting collecting water with a friendly companion or having a chat at the well, etc. (see [17]). Technical innovations, such as the use of multiple hand pumps attached through lateral pipes to a single borehole, may help overcome distance barriers. Naturally, the installation of greater numbers of deep tubewells will also reduce the distance from users and hence the inconvenience.

**Household arsenic removal** **technologies** score high in terms of acceptance. Only two negative issues were revealed: low descriptive norm, i.e. users do not perceive that others also use household arsenic-removal, and difficulty to remember filtering water at the right time. These issues can be overcome, for example, by pointing out other households that use arsenic removal filters, and posting graphical reminders at the location of the filter [18].

**Community arsenic removal technologies** reach a medium level of acceptance, except that the norm factors are above average. This means that users perceive many others who collect water from this source and that others appreciate their use. Users and non-users, however, rate the temperature of the water from this option as undesirable. A potential intervention could be to instruct people to cool the treated water via storage in clay pots or wrapping wet fabric around the containers.

**Pond sand filters** also reach a medium level of acceptance, but are considered to be time-consuming, and temperature seems to be a problem. Users of pond sand filters, in addition to issues with water temperature and time, face difficulties of self-efficacy; people are not sure they can collect all the water they need from this option. To increase self-efficacy, further information must be collected regarding where the problem lies. For example, if it is a matter of not having enough people to collect enough water, other households may be prompted to collect water together. However, if it is a malfunction of the filter, the device needs to be improved or further water points need to be implemented.

**Rainwater harvesting** also has only a medium level of acceptance. Although users and non-users both think that rainwater harvesting is not time-consuming, this option scores particularly low in terms of normative factors. This means that people do not perceive many others to be using this option and that few others appreciate their use. Therein may lie the reason why rainwater is collected by only a few households. A possible intervention to enhance social norms could be to convince well-known persons to praise this option.

**Dug wells** and **well-sharing** score the lowest on acceptance; most of the psychological factors are below average. Both options are rated as being particularly time-consuming. Well-sharing is additionally low in the injunctive norm, implying that others do not appreciate the use of this option. For both options, users do not see alternatives that could be used to overcome their barriers (low levels of coping planning), and they are not confident they will get as much water as needed (low levels of self-efficacy). This all results in users’ low commitment, indicating that if there were another safe water option, these users would change immediately.

Users of both dug wells and well-sharing also reported dissatisfaction with the temperature, taste, and odor of drinking water. The poor taste of water from dug wells is consistent with its relatively poor microbiological quality [33]. Household water treatment might improve both the taste and quality; adding a few drops of citrus juice could improve the taste. As noted earlier, clay containers could cool water during warm seasons. If there are no practical alternatives to dug wells or well-sharing, then households should plan which other wells can be used if the one they normally use becomes inaccessible. Agreements with the owners of these alternative wells should be arranged. The householders could then feel more confident in collecting as much safe water as they need and feel more committed to using safe water.

In contrast with its psychological ratings, **well-sharing** emerged as one of the most used options in this study. This is consistent with previous estimates [13]. It is therefore surprising that well-sharing scored low in terms of psychological factors. This may indicate that people with access to neighboring safe wells do perceive this as the only available safe water option and therefore use it. It seems that well-sharing is perceived as only a temporary solution: if other safe options were installed in the neighborhood, households would most likely prefer these over well-sharing. Furthermore, due to the lack of support from the psychological factors, it seems likely that well-sharers switch back to using their contaminated wells at least occasionally. These assumptions require further investigation.

### Strengths and Limitations

A particular strength of the present study was the comprehensive theoretical framework employed to investigate the acceptance of arsenic-safe water options. Furthermore, to our knowledge, this is the first study that systematically investigated the acceptance of all commonly implemented arsenic-safe water options in Bangladesh. This overview provided valuable insights on the use and acceptance of arsenic-safe water options and had implications for arsenic mitigation. However, some limitations must also be addressed.

First, our results may be positively biased for household arsenic removal due to the high survey refusal rate: A third of the households listed as having received filters denied ever having received a filter and were therefore not interviewed. This indicates poor acceptance of household filters by at least a part of the population, which is not reflected in our results.

Furthermore, participants with access to different arsenic-safe water options differed regarding their monthly incomes. These income differences may have explained the different use levels of the safe water options. However, as was indicated by multivariate analyses, income was not significantly different between users and non-users overall, or between users and non-users of any particular option. This indicates that the differing use levels of the water options are not attributable to differences in income. A further source of bias may be differences in the number of households who paid for installing the arsenic-safe option, and differences in religion. Regarding payments, results indicate that more users had paid for installing the safe option than non-users. Therefore, higher acceptance of options may have been confounded with higher numbers of people who paid for installing these. However, when comparing with the average number of users who had paid for installation, piped water users, arsenic removal filter users, and rainwater users showed higher numbers of people who had financially contributed. As the latter showed very low acceptance, such a bias is highly unlikely. Results on religion, on the other hand, indicate that a smaller proportion of users were Muslims than non-users. However, a religion bias seems unlikely, as the most accepted options (piped water supply and deep tubewells) showed above average proportions of Muslims.

The measurement instrument employed here may have had shortcomings. Although the RANAS factors were operationalized in accordance with the model’s specifications, mostly one-item scales were used to operationalize them. The single items were typical questions for the respective construct selected from a larger item pool that had been analyzed by factor analysis. Thereby, the validity of the items and constructs was ascertained. This approach was chosen to keep the analyses simple and understandable for a broad audience.

Further regarding measurement, households with access to deep tubewells showed greater action knowledge than the average household prone to arsenic contamination. However, this is most likely attributable to the fact that action knowledge was assessed differently in the deep tubewell study than in the 2009 study, when all other arsenic-safe water options were investigated. Further studies should employ consistent knowledge measures and investigate this further.

Finally, this was the first application of the arsenic-framed, Bengali version of the questionnaire, which may be seen as a limitation. However, careful translation procedures and extensive pretesting provide strong confidence in the applicability of the standard questions for the local context.

### Conclusions

This study provides insights into people’s differential acceptance of all of the arsenic-safe water options commonly promoted in Bangladesh. For each safe water option, psychological factors have been identified that should be improved in order to increase their acceptance. To maximize the impact of arsenic mitigation efforts, greater emphasis should be given to the installation of psychologically-favored options (deep tubewells and piped water supply) rather than poorly supported options (dug wells, pond sand filters, and rainwater harvesting). Well-sharing is also poorly supported and should be seen as only a temporary solution. Regardless of the technology promoted, an understanding of the key underlying psychological factors described in the RANAS model can be used to develop interventions tailored to influencing the relevant drivers of behavior change. This type of ‘smart’ intervention, coupled with sound technologies, has the potential to improve the efficiency of arsenic mitigation efforts.
